# For the Best-equipped Meat-inspectors

**Published:** 1898-02

**Authors:** 


### FOR THE BEST-EQUIPPED MEAT-INSPECTORS.

We most heartily concur in the following opinion, voiced by Dr. C. W. Stiles in an address before the Maryland Medical Society, and we are sure that much good will flow from the thorough investigation made by Dr. Stiles of many private slaughter-houses in our cities and towns:

"My second suggestion relates to the director of the proposed municipal abattoir. If the slaughter-house is placed under city control, the natural tendency among certain people will be to claim that patriotism calls for the appointment of the director according to his political pull. I would modestly suggest that an unsuccessful blacksmith, or barber, or a physician, dentist, or druggist who has failed in your State examination is hardly the person to be appointed director of a municipal abattoir, notwithstanding his political pull. In this matter of public health we are dealing with life and death, and we must have a man equal to the position. Personally, I believe that the man appointed should be a veterinarian, and should be, ex-officio, a member of the local board of health. And by this word veterinarian I do not mean a quack horse-doctor, but rather a well-educated and scientifically trained graduate of a reputable school, and, besides that, a man of experience in gross pathology and meat-inspection. The scientific meat-inspector can render public service in the prevention of disease among men and live-stock to a degree scarcely dreamed of by the non-technically trained laity."

Every chief magistrate or executive officer of our cities should be acquainted with the results of the investigations made in this direction by Dr. Stiles, and we are sure that no better line of action could be adopted by veterinarians than to place the same before such officers and all boards of health.

